# Synchrony to a beat predicts synchrony with other minds

**DOI:** 10.1038/s41598-023-29776-6

**Published:** 2023-03-03

**Authors:** Sophie Wohltjen, Brigitta Toth, Adam Boncz, Thalia Wheatley

**Affiliations:** 1 Psychological and Brain Sciences Department, Dartmouth College, 6207 Moore Hall, Hanover, NH 03755 USA; 2 Psychology Department, University of Wisconsin, 1202 West Johnson St. Madison, Madison, WI 53706 USA; 3 Institute of Cognitive Neuroscience and Psychology, Research Centre for Natural Sciences, Magyar Tudósok Körútja 2, Budapest, 1117 Hungary; 4 Santa Fe Institute, Santa Fe, NM USA

**Keywords:** Psychology, Human behaviour

## Abstract

Synchrony has been used to describe simple beat entrainment as well as correlated mental processes between people, leading some to question whether the term conflates distinct phenomena. Here we ask whether simple synchrony (beat entrainment) predicts more complex attentional synchrony, consistent with a common mechanism. While eye-tracked, participants listened to regularly spaced tones and indicated changes in volume. Across multiple sessions, we found a reliable individual difference: some people entrained their attention more than others, as reflected in beat-matched pupil dilations that predicted performance. In a second study, eye-tracked participants completed the beat task and then listened to a storyteller, who had been previously recorded while eye-tracked. An individual’s tendency to entrain to a beat predicted how strongly their pupils synchronized with those of the storyteller, a corollary of shared attention. The tendency to synchronize is a stable individual difference that predicts attentional synchrony across contexts and complexity.

## Introduction

An enormous literature explores human synchrony across a wide array of behaviors, contexts, and timescales. People not only synchronize to a simple beat (commonly referred to as entrainment) but also to more complex stimuli that vary continuously over time, such as musical scores^1,2^, natural speech^3^, and even the physiological dynamics of other people. Across these various domains, synchrony is computed in different ways and at different timescales, leading some to wonder whether the term itself has any explanatory precision^4^. Is “synchrony” at one level predictive of “synchrony” at another, or does the term conflate disparate phenomena? Here we ask whether simple beat entrainment predicts complex attentional synchrony within the same individual, consistent with a common mechanism.

Recent genetic and neurological evidence suggests that the tendency to synchronize may be a stable individual difference, at least to a simple beat. Niarchou and colleagues^5^ demonstrated that people are able to accurately report whether they can “tap in time to a musical beat.” Moreover, this ability is polygenic, underpinned by a genetic signature linked to several biological rhythms such as breathing, walking pace, and circadian chronotype. A large study of 5648 Swedish twins also found that beat synchronization ability in a finger-tapping task runs in families^6^. Further consistent with a stable, neurological basis, some neurological disorders affect synchrony. For example, Parkinson’s disease is thought to impair a beat-based timing mechanism^7^ that implicates the same cortical circuits underlying individual differences in rhythm production^8^. Individuals with attention deficit hyperactivity disorder (ADHD) struggle with sensorimotor synchronization to isochronous beats^9^, which may stem from impairments with sustained attention^10^. Children with autism spectrum disorder (ASD) also have difficulty synchronizing their movements to a simple rhythmic stimulus, both intentionally^11^ and spontaneously^12,13^. This led Marsh et al.^13^ to conclude that “deficiencies in perceiving and responding to the rhythms of the world may have serious consequences for the ability to become adequately embedded in a social context” —a statement consistent with a later study finding that individuals with ASD were also less likely to synchronize their movements in real-world social interactions which are “seldom perfectly rhythmic but rather highly complex and unpredictable”^14^. Collectively, these studies suggest that the tendency to synchronize may vary between individuals (as genes and disorders do) yet be stable within an individual, across levels of complexity.

It is possible to measure synchrony without requiring motor responses. It is well-established that, under light constant conditions, pupil dilations index attention^15,16^ and that attending to rhythmic stimuli causes the pupil to dilate and constrict in synchrony (e.g., along with visual oscillations^17^ and auditory beats^18^). Although the neurobiological circuitry causing this link remains unclear, pupil dilations are temporally coupled to neural firing in the locus coeruleus^19,20^ associated with exploratory behavior and guiding attention^21–23^. Pupil dilations therefore provide a temporally sensitive, implicit, and easily measurable index of attention as it changes over time^24–27^.

Pupillary dilations also track conscious attention to more complex, naturally varying stimuli. For example, when hearing two pieces of music dichotically (a different piece playing, simultaneously, in each ear) but *attending* to only one ear, one’s pupil dilations are more similar to the way their pupils respond when listening to that piece of music alone^2^. And when people attend in the same way at the same time, their pupil dilations synchronize thus providing a dynamic trace of when two people are sharing attention^28–30^. As shared attention predicts, interpersonal pupillary synchrony is particularly strong between listeners during emotionally salient moments of stories and between interlocutors during engaging conversations. By virtue of being an implicit and dynamic metric of attention measurable in both isochronous and naturally varying rhythms, pupil dilations afford a test of whether the tendency to “get in sync” manifests across these different levels of complexity within the same individual.

Measuring synchrony using pupil responses not only allows for the tracking of synchrony across levels of complexity within the same individual, it also provides a test of potential underlying cognitive processes. Because pupillary entrainment is thought to reflect predictions about statistical regularities in the environment^31^, one possible mechanism involved in diverse forms of synchrony may be predictive coding. The tendency to mind-wander, a state associated with decreased sensitivity to attention tasks^32^ and decreased sensorimotor synchronization^33^ has also been linked to pupillary decoupling^25^, suggesting that more general attentional states could also be at play. To untangle the potential influence of these factors, we modified the well-established oddball task. Specifically, target (oddball) tones were presented at predictable intervals and novel tones were presented at unpredictable intervals. This allowed us to test whether pupillary entrainment is enhanced by predictive structure beyond a general attentiveness to all stimuli.

In Study 1, we used this modified oddball paradigm to test whether individuals’ pupil responses to auditory events, including the tendency to synchronize to the beat of rhythmically presented tones, comprised a reliable individual difference. In Study 2, we again used the oddball paradigm to test whether individual differences in pupillary entrainment synchrony predicted differences in (1) oddball task performance, and (2) the degree to which people synchronized their attention with the attention of another person during a dynamic, naturalistic listening task.

## Study 1: Are there reliable individual differences in pupillary entrainment synchrony?

In Study 1, we examined whether individuals reliably differed in the degree to which their pupil dilations synchronized with the beat of rhythmically presented tones in an oddball task.

## Method

### Materials

#### Participants

Eight participants (mean age: 20.13; 5 females) each participated in nine, 30-min experimental sessions. Participants were recruited from Dartmouth College and were compensated with extra credit in a college course. All participants had normal or corrected-to-normal vision. The ethics committee for the Protection of Human Subjects (CPHS) at Dartmouth approved all study procedures, and written informed consent was obtained prior to the start of the study. All methods were performed in compliance with the guidelines and regulations set forth by CPHS.

#### Eye-tracking data collection

Pupil dilation data was collected continuously while participants completed the task. Pupil diameter was recorded from both eyes at 30 Hz using SensoMotoric Instruments (SMI) wearable eye-tracking glasses (SensoMotoric Instruments, Teltow, Berlin). Participants placed their head in a chin rest located one foot from a computer in a luminance-controlled testing room. Lux values were recorded at multiple points in the room to ensure that luminance in the room did not exceed the 150-lx necessary to elicit a luminance-induced pupil dilation^34^.

### Procedure

#### Oddball detection task

To obtain a repeated measure of participants’ pupil responses, each of eight participants completed the task nine times. All participants finished all nine sessions within four weeks.

Participants were presented with a series of auditory tones while instructed to keep their eyes on a dot located at the center of the computer screen. They were told that some of the tones they heard would be quieter than the others (the “oddball” tones), and they were instructed to press the spacebar on a computer keyboard anytime they heard one of the quieter tones. Each tone was presented for 50 ms, and tones were spaced 1200 ms apart. The entire experiment consisted of five blocks of 160 tones each. Tones were organized into four experimental conditions: standard, target, novel, and omission. “Standard” tones were sine wave pure tones with a frequency of 880 Hz and the most commonly heard, comprising 65% of all trials. Target (oddball) tones were quieter (individually thresholded—see supplementary materials for more details) versions of the standard tones and comprised 20% of total trials. Because standard and target trials happened repeatedly at perfectly predictable intervals of 1200 ms, and because target trials were nested within this 1200 ms-interval structure (occurring predictably at every fourth trial in this sequence, i.e., every 4800 ms), these two trial types comprised a test of whether an individual’s pupils would entrain to the temporal structure of the task. This spacing between target trials ensured that participants’ pupils would have sufficient time to return to baseline in between target presentations.

Another 10% of the trials were “novel” tones created by adding different frequencies to the standard 880 Hz pure tone. These frequencies were randomly selected using a random number generator, from frequency values equally spaced on a logarithmic scale from 1320 to 5280 Hz. Novel trials could occur at any point in the sequence and were thus not predictable. These trials therefore comprised a test of whether reactivity alone would predict behavior.

**Figure 1:**
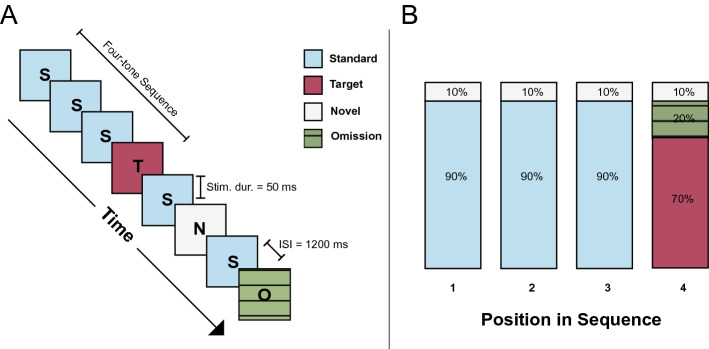
(**A**) Schematic of the auditory oddball paradigm. Auditory stimuli were tones presented for 50 ms each, with an inter-stimulus interval of 1200 ms. Each tone belonged to one of four stimulus categories—standard, target, novel, and omission. Participants were instructed to press the spacebar whenever they heard a target tone. All stimuli were grouped into larger sequences comprising four tones. (**B**) shows the percentage of time that stimuli from each category appeared at each position in the four-tone sequence. Figure created using Adobe Illustrator (Adobe Inc., 2020; Retrieved from https://adobe.com/products/illustrator).

The remaining trials were omission trials which occurred at the position of expected target tones and therefore were designed to evoke a prediction error. These trials assessed reactivity to prediction error. These omissions comprised 20% of tones occurring on the fourth beat, and 5% of tones overall. An illustration of the experimental setup and stimuli distributions can be found in Fig. 1.

### Data analysis

#### Preprocessing and quality control

Raw pupil dilation time series were preprocessed for further analysis by first performing linear interpolation over eye-blinks and other drop-out in signal. To remove spikes, the data was median filtered (5^th^ order) and low-pass filtered at 10 Hz. we then separated the pupil dilation time series into three second trials and preprocessed according to the guidance of Mathôt, et al.^35^, removing any trials that contained pupil sizes smaller than 2 mm. The remaining trials were z-scored and baseline-corrected by subtracting (rather than dividing; see^35^) the mean pupil size of the 100 ms prior to each individual trial from the trial data. For 16 (of 72 total) sessions, pupil dilations were not recorded for a large portion (> 90%) of the experiment due to technical issues, and thus were not included for further analysis.

#### Average pupil responses, per condition

**Figure 2:**
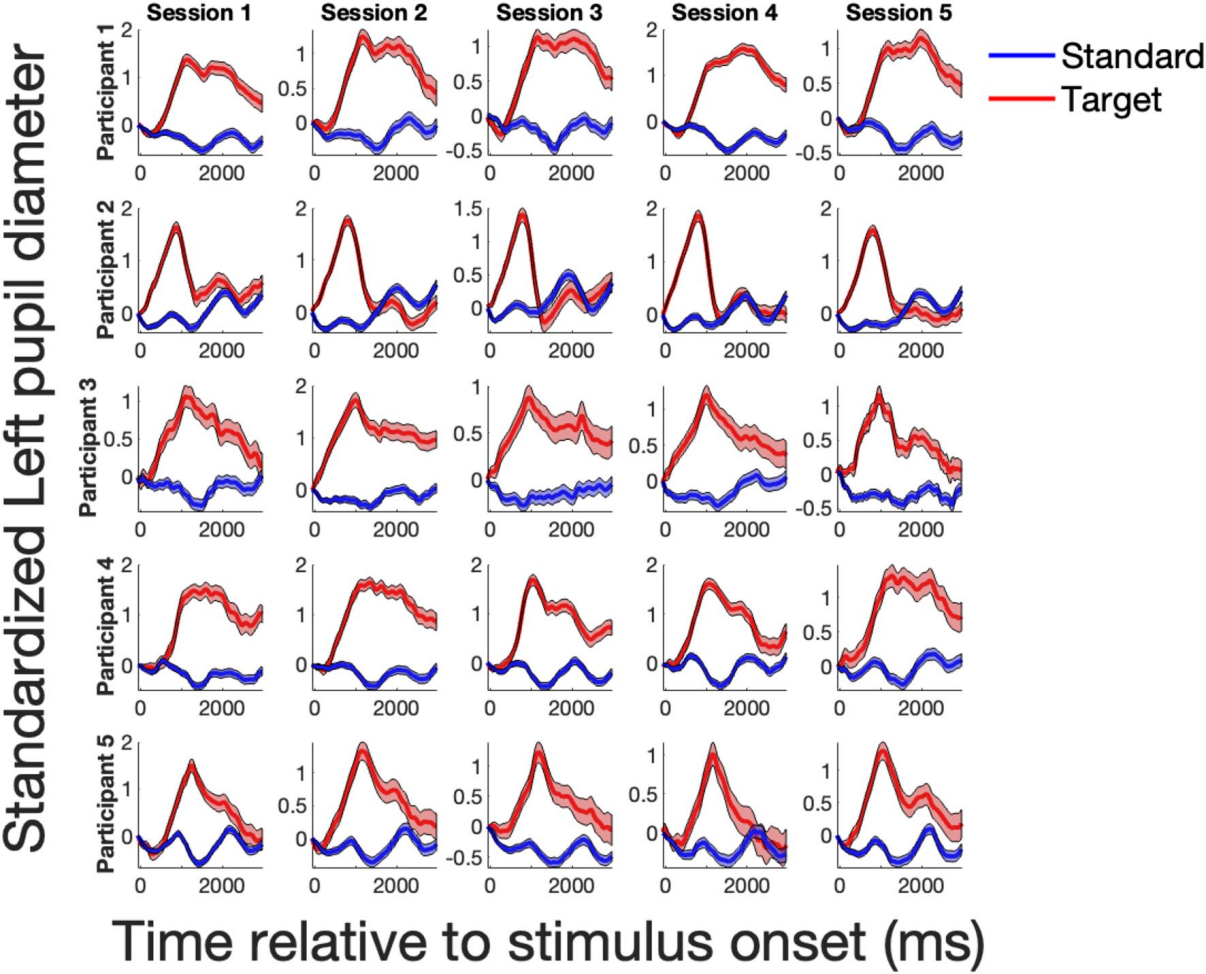
Illustration of pupil response curves over five experimental sessions for five participants. Each row depicts pupil responses for a single participant. Each column shows a different experimental session. Pupil responses to the target tone are shown in red, and pupil responses to the standard tone are also plotted for comparison and shown in blue. Response curves were different between participants, but highly reliable within participants.

We first calculated the average pupil response to all four tone categories from each session per participant. For target (oddball) tone trials, we averaged pupil responses for correct trials. An illustration of these final pupil response curves for five of our participants can be found in Fig. 2.

#### Entrainment synchrony

“Entrainment” is a form of synchrony that denotes an individual moving in sync with some externally generated, rhythmic oscillation^36^ Previous research has demonstrated entrainment of pupil dilations to both visual^17^ and auditory^18^ stimuli. This pupillary entrainment is thought to reflect attention^17,18^ as well as predictions about statistical regularities in the environment^31^. In the oddball paradigm there were two predictable intervals to which participants could entrain: the inter-stimulus interval (ISI; 1200 ms, or 0.83 Hz) as well as the target-stimulus interval (TSI; 4800 ms, or 0.21 Hz). To assess participants’ propensity to entrain, we extracted the continuous, raw pupillary signal for each of the five blocks of the oddball task. We then preprocessed each block by interpolating over eye blinks using a cubic spline interpolation and using a spectral power analysis to determine how tightly coupled participants were with the ISI and TSI. Spectral power analysis allows for the decomposition of a time series into *frequencies,* corresponding to oscillations at particular intervals, and *power*, corresponding to the squared amplitude of each frequency in the time series. Higher power for a given frequency component represents a stronger oscillation at this interval. We windowed each block with a Hamming function and estimated power using a periodogram. Because we were interested in comparing power at the ISI and TSI across participants, we standardized each participant’s frequency spectrum by computing a z-score of the power around the ISI (0.8–0.9 Hz) and the TSI (0.2–0.3 Hz). These resulting power values were then summed and are from now on referred to as “entrainment synchrony” scores.

## Results

### Pupil responses reflect reliable differences in an individual’s tendency to synchronize

To determine whether participants reliably differed in their tendency to synchronize their continuous pupillary fluctuations to the rhythmic interval of the tones in the oddball task, we first established that participants showed individually specific and reliable pupil responses to standard and target trials. Figure 2 illustrates the within-participant similarity for target and standard tones.

We tested the similarity of participants’ averaged pupil responses in each of the trial-types of the oddball experiment across testing sessions. Pearson correlations compared participants’ pupil response curves in each session to their own responses from different sessions. We then compared the resulting R values from those correlations to the R values obtained by correlating pupil responses between participants. Because our data were not normally distributed (within-participant correlations were skewed toward higher R values) and because we had more between-participants observations than within-participants observations, we used the non-parametric Mann–Whitney U test to compare pupil response similarity within and between participants.

**Figure 3:**
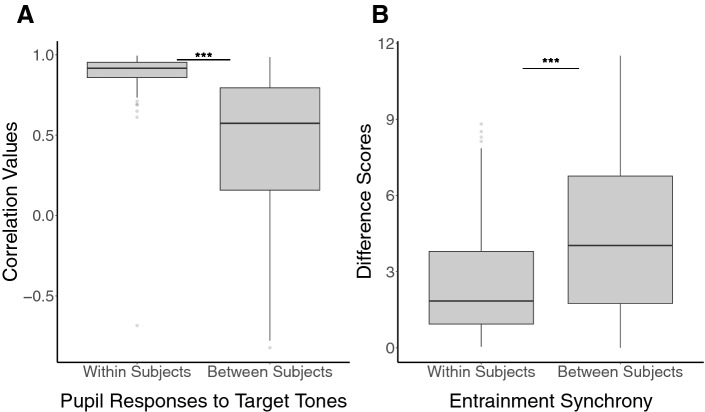
(**A**) Boxplot comparing correlations of pupil response curves to target tones within and between participants. Within-participants correlations (M = 0.89, SD = 0.15) were significantly higher than between-participants correlations (M = 0.44, SD = 0.44; *p* < 0.001), suggesting that pupil responses for a single individual are both different from other individuals’ and reliably similar across multiple experimental sessions. (**B**) Boxplot comparing difference scores of pupillary entrainment synchrony across the oddball task within and between participants. Within participants difference scores (M = 2.6, SD = 2.18) were significantly lower than between participants difference scores (M = 4.39, SD = 2.97; *p* < 0.001), suggesting that the intensity with which an individual synchronizes with the temporal dynamics of the oddball task is reliably similar across multiple experimental sessions and different from other individuals.

Participants’ pupil response curves to target tones were more similar to themselves across sessions than compared to other participants (U = 151,877, *p* < 0.001, Cohen’s *d* = 1.37, Fig. 3A). Further, we found that this reliability was not specific to target tones but was also found for participants’ pupil responses to standard tones (U = 138,465, *p* < 0.001, Cohen’s *d* = 1.06). Pupil responses to novel and omission conditions were also reliable within participants, across testing sessions. Complete results for these conditions can be found in supplementary materials.

To investigate whether participants also synchronized with the temporal structure of the oddball task in a way that was distinct and reliable across testing sessions, we compared difference scores obtained by subtracting a sum of participants’ entrainment synchrony scores at the ISI and TSI (see methods for more details on entrainment synchrony calculation) from their own entrainment synchrony during different sessions. We then compared the resulting difference scores to the difference scores obtained by subtracting entrainment synchrony between participants. Because our data was not normally distributed (within-participant difference scores were skewed toward lower differences) and because we had more between-participants observations than within-participants observations, we again used the non-parametric Mann–Whitney U test to compare entrainment similarity within and between participants.

Participants’ entrainment synchrony score in any given session was more similar to their other sessions than compared to other participants (U = 55,493, *p* < 0.001, Cohen’s *d* = −0.69, Fig. 3B). This demonstrates that individual pupillary entrainment synchrony is a reliable individual difference.

In Study 1, we show clear evidence for reliable pupil responses that reflect an individual’s tendency to synchronize with the temporal dynamics of the oddball task. Study 2 asked whether this tendency is also (1) behaviorally meaningful and (2) transferable across contexts. Specifically, study 2 examined whether an individual’s tendency to synchronize with an externally generated, rhythmic oscillation is predictive of performance in the same psychophysics paradigm of Study 1 (detecting an oddball tone) and whether this tendency toward entrainment synchrony transfers to an individual’s tendency to synchronize their continuous pupillary fluctuations with the pupil dynamics of another person in a separate, more unstructured listening task.

## Study 2: Are individual differences in pupillary entrainment synchrony behaviorally meaningful and transferable to different, more complex forms of synchrony?

To investigate whether an individual’s tendency to synchronize is behaviorally meaningful as well as transferable to different forms of pupillary synchrony, we examined participants’ pupil dilations across two tasks: the highly-structured oddball task in which stimuli are presented rhythmically, and a highly-variable task in which the participant listens to speakers recounting emotional memories.

## Method

### Materials

#### Participants

82 participants (mean age:19.9; 46 females) were recruited from Dartmouth College and were compensated with extra course credit for participation. All participants had normal or corrected-to-normal vision. The ethics committee for the Protection of Human Subjects (CPHS) at Dartmouth approved all study procedures, and written informed consent was obtained prior to the start of the study. All methods were performed in compliance with the guidelines and regulations set forth by CPHS.

#### Eye-tracking data collection

Eye-tracking data collection followed the procedure outlined in Study 1.

### Procedure

#### Oddball detection task

The paradigm was identical to Study 1 with the exception that participants in Study 2 only completed one session of the task instead of nine.

#### Listening task

**Figure 4:**
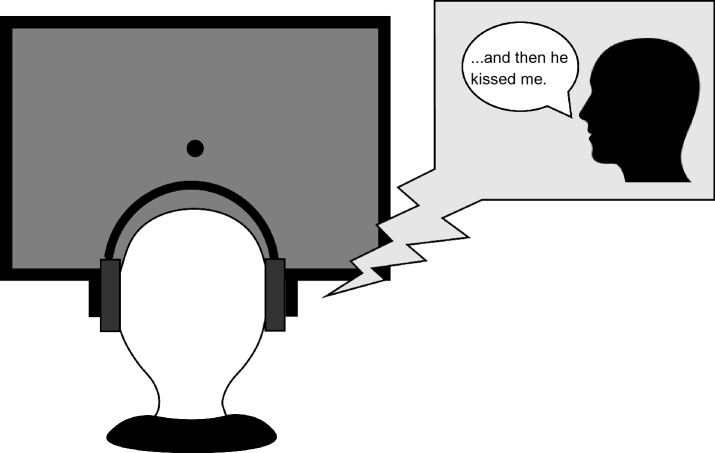
Illustration of the listening paradigm. Participants listened to speakers telling emotional stories while they fixated on a dot in the center of a computer screen. Both speakers’ and listeners’ eyes were tracked during this task. Figure created using Adobe Illustrator (Adobe Inc., 2020; Retrieved from https://adobe.com/products/illustrator).

Participants placed their head in a chin rest located one foot from a computer screen. For this task, participants’ eyes were tracked while they listened to four personal, emotional stories told by highly expressive speakers. These speakers were determined to be highly expressive based on their scores on the Berkeley Expressivity Questionnaire, which assesses three different facets of emotional expressivity—negative expressivity, or how expressive one is when communicating negative emotions like anger or sadness, positive expressivity, or how expressive one is when communicating positive emotions like happiness, and impulse strength, or how strongly one feels their emotions internally^37^. These speakers’ recorded stories and pupillary time series were originally collected for use in Kang & Wheatley’s^28^ paper on pupillary synchrony between speakers and listeners. These speakers told their stories while their left eye was tracked at 120 Hz using an ASL Eye-Trac 6 eye tracker. The order of these stories was randomized, and participants were instructed to watch a dot on the center of the computer screen while they listened, in order to control for potential pupillary effects caused by shifts in gaze. Listener’s eyes were also tracked, using SMI wearable eye-tracking glasses (SensoMotoric Instruments, Teltow, Berlin). A visual of this experimental setup can be found in Fig. 4.

### Data analysis

#### Preprocessing and quality control

For pupillary time series collected during the oddball task, raw pupil dilation time series were preprocessed for further analysis by first performing linear interpolation over eye-blinks and other drop-out in signal. To remove spikes, the data was median filtered (^5th^ order) and low-pass filtered at 10 Hz. For analyses that included trial-level responses, we then separated the pupil dilation time series into three second trials and preprocessed according to the guidance of Mathôt, et al.^35^, removing any trials that contained pupil sizes smaller than 2 mm. The remaining trials were z-scored and baseline-corrected by subtracting (rather than dividing; see^35^) the mean pupil size of the 100 ms prior to each individual trial from the trial data. After preprocessing and removing participants for whom pupil dilations were not recorded for a large portion (> 90%) of the experiment, 72 participants (M age 20.16, 39 females) were included for further analysis.

Unlike event-related designs, analyses using the entire pupillary time series cannot benefit from averaging over many trials to ensure an appropriate signal to noise threshold. For this reason, for pupillary time series collected during the listening task, participants were removed who required more than 25% of their data interpolated due to eye-blinks and other dropout in signal (per^28^). To remove spikes, the data was median filtered (5th order), low-pass filtered at 10 Hz, and detrended to reduce drift. After preprocessing and removing participants with more than 25% missing data, 63 participants (M age 20.12, 34 females) with both usable oddball and usable listening data were included for further analysis.

#### Task performance

To assess participants’ performance during the oddball task, we calculated a *d* prime value for each participant using the R package “psycho”^38,39^. *D* prime is a widely used measure of sensitivity that considers both the number of correct target identifications (here, spacebar presses for target tones) compared to the overall number of targets, as well as the number of false alarms (here, spacebar presses for anything other than a target tone) compared to the number of distractors. The formula for calculating *d* prime is as follows:

$$d^{\prime} \, = \, Z\left( {hits \, / \, n \, targets} \right) \, - \, Z\left( {false \, alarms \, / \, n \, distractors} \right)$$

#### Entrainment synchrony

Pupillary entrainment synchrony during the oddball task was calculated in the same way as in study 1.

#### Dynamic time warping

We used Dynamic Time Warping (DTW) to measure synchrony between speakers’ and listeners’ continuous pupillary time series. DTW is an algorithm used to compare signals that may be offset in time^40^. The DTW algorithm divides two signals into a user-defined number of segments, each representing some window of time. Then, DTW calculates the cosine similarity of these segments. DTW makes three comparisons for each segment: the same segment on both signals, and that segment on one time series with the following segment on the other. The cosine similarity values associated with these three segment pairs are compared. The segment pair that yields the smallest cosine similarity value is deemed to be the time at which the content of the two signals best align, and both signals are adjusted according to that cosine similarity comparison. Each adjustment incurs a penalty, or a ‘‘cost’’ of realignment. The sum of these penalties yields an overall cost value, which represents the overall effort involved in warping one signal onto the other. Higher cost values indicate greater dissimilarity between two patterns.

## Results

### Individual differences in pupillary entrainment synchrony predict oddball detection.

**Figure 5:**
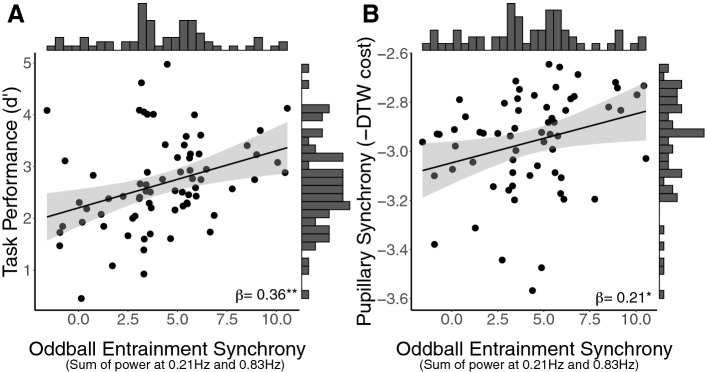
Data and estimated slopes for the relationship between (**A**) Oddball task performance and a sum of relative power at the inter-stimulus interval (ISI) and target stimulus interval (TSI). This relationship suggests that participants who performed the oddball task more accurately (as measured by *d* prime) were also more synchronized with the oddball task structure. (**B**) Relationship of pupillary synchrony between speakers and listeners with oddball entrainment synchrony. Entrainment synchrony was also positively related to pupillary synchrony such that the more synchronized participants were with the beat during the oddball task, the more synchronized their pupils were with the speaker in the listening task.

To test whether pupillary entrainment synchrony predicted oddball task performance, we ran a linear regression predicting individual task performance from the sum of each individual’s entrainment synchrony at the ISI and TSI. This model was significant (f(1,70) = 10.72, *p* = 0.002, adjusted R^2^ = 0.12), with entrainment positively predicting *d*’ task performance (β = 0.36, *p* = 0.002). This suggests that individual differences in an individual’s tendency to synchronize are not only reliable, they also predict differences in these individuals’ behavior during the oddball task. Illustrations of this result can be found in Fig. 5A.

Because entrainment synchrony is hypothesized to reflect continuous predictions about statistical regularities^31^, implicit learning, and anticipatory attending^41–43^, we also analyzed trial-level pupil responses to the novel and omission trials. The novel trials are events in the absence of prediction whereas the omission trials reflected prediction in the absence of an event (i.e., the expected but missing target tone; see Study 1 methods for a detailed description of these conditions). For *novel* trials, pupil responses were not predictive of task performance (f(3,68) = 1.11, FDR corrected *p* = 0.35, adjusted R^2^ = 0.004), or entrainment synchrony (f(3,68) = 0.56, FDR corrected *p* = 0.77, adjusted R^2^ = −0.02) consistent with entrainment synchrony reflecting prediction-guided attention rather than a general increase in attentiveness or heightened reactivity.

However, *omission* trials, which were also unexpected but, unlike novel trials, occurred at predictable locations in the sequence, did significantly predict task performance (f(3,68) = 3.53, *p* = 0.02, adjusted R^2^ = 0.1) and marginally predicted entrainment synchrony (f(3,68) = 3.52, *p* = 0.07, adjusted R^2^ = 0.06), with the *amplitude* of the omission trial pupil response significantly driving both of these effects (larger pupil responses to omission trials were predictive of task performance; β = 0.38, FDR corrected *p* < 0.005, and entrainment synchrony; β = 0.27, FDR corrected *p* = 0.04). This finding suggests that those individuals who were more synchronized with the tones in the oddball task were doing so as a result of predictive attention based on encoding the structure of the task, rather than a more global task motivation or general attentiveness. Full details of this analysis can be found in supplementary information.

### Individual differences in oddball entrainment synchrony predict pupillary synchrony between speakers and listeners.

As a measure of attentional synchrony, we calculated pupillary synchrony between speakers and listeners in three-second windows with 1.5 s of overlap, then averaged these into six-second windows, and finally log-transformed to correct for homoscedasticity (per^28^). We then averaged resulting synchrony scores over each story, such that each participant had one overall pupillary synchrony value for each of the four stories they heard. We ran a linear-mixed model predicting participants’ pupillary synchrony with each speaker from their entrainment synchrony during the oddball task. Participants and stories were entered into the model as random effects. We found a significant main effect of entrainment synchrony (t(61.72) = 2.13, β = 0.21, *p* = 0.04), such that participants who were better at synchronizing with the task structure during the oddball task also showed higher levels of pupillary synchrony with the speaker during the listening task. Illustrations of this result can be found in Fig. 5B.

**Figure 6:**
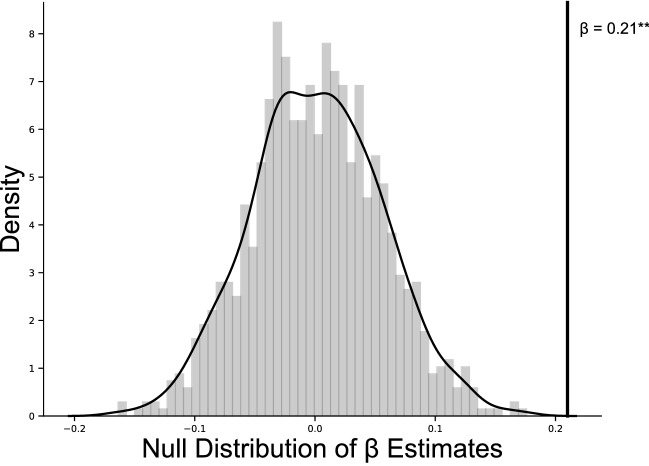
Results of a permutation test comparing the true estimate (β = 0.21) of the association between entrainment synchrony and speaker-listener pupillary synchrony to 1000 permutations shuffling participants’ entrainment synchrony values. The true relationship between entrainment synchrony and speaker-listener pupillary synchrony fell significantly outside these two distributions.

To more robustly test the probability that our effect of entrainment synchrony on pupillary synchrony was not due to chance, we performed a permutation test where we compared our true effect estimate to a null distribution created by shuffling participants’ entrainment synchrony values. Specifically, we shuffled participants’ entrainment synchrony values, then ran the same linear mixed model described above on this shuffled data. We repeated this 1000 times to build our final null distribution to compare to our true effect. Our true effect was significantly positively skewed from this distribution (*p* < 0.001), again suggesting that an individual’s task entrainment synchrony is related to their dyadic pupillary synchrony above chance. A visualization of the null distribution created by these permutations in relation to the true relationship between entrainment synchrony and pupillary synchrony can be found in Fig. 6.

## Discussion

Here, we present evidence for reliable, individual variation in the tendency to synchronize attention across stimuli from highly periodic beats to the continuously varying dynamics produced by another mind. Individuals whose pupils were more tightly synchronized with the rhythmic sequence of the oddball task were also more likely to synchronize their pupillary dilations with those of an individual telling a story, a corollary of shared attention^28,30^. These results suggest that the tendency to couple one’s attention to external stimuli is a reliable individual difference that varies in the human population and manifests across levels of complexity.

From the first discovery of attention-linked pupillary responses, scientists have tended to average the pupillary signal across participants. This approach amplifies the signal common across evoked stimulus responses and has yielded a number of important findings. For example, averaging across participants revealed that pupil dilations tend to track cognitive effort^16^, arousal^44^ and attention^27,45^. But such averaging, by definition, obscures potentially meaningful variation between people, assuming instead a kind of canonical, average person. Here we show that there is no canonical level of synchrony. Instead, the tendency to synchronize one’s attention varies widely between individuals. We further show that, within an individual, this tendency is stable over time (multiple testing sessions), context (vigilance task and story listening) and complexity (rigid periodicity and continuously varying stimuli).

Given the association between pupil dilations and attention, the finding that pupils entrain to an attended, periodic stimulus is not surprising and replicates previous research^18^. It is less obvious why this simple form of entrainment synchrony predicts more complex attentional synchrony. As listeners never saw the speakers, visual entrainment (i.e., pupil mimicry ^46^;) can be ruled out. Instead, these findings suggest that both paradigms (oddball and storytelling) provide dynamic information streams to which attention can entrain. In the oddball task, the information stream was highly regularized and predictable: simple, regular beats with every fourth beat the location of a possible change (oddball or omission trial). Responses to these changes on the fourth beat were associated with an individual’s level of beat entrainment, while responses to novel sounds at unpredictable intervals were not. This suggests that the tendency to entrain is more about a sensitivity to predictive structure than about having a more global, heightened attentional state. In the storytelling task, the information stream was complex, semantic and variable, yet likely leveraged predictive structures of speech^3,47,48^ and narrative^49^, as well as attentional devices such as prosodic cues to salient information^50,51^. These devices are most apparent in infant-directed speech, which tends to have rhythmic features that help orient the infant to salient semantic information^52^. In adult speech, these features are deployed less often and more sporadically, with fluctuating periodicities facilitating expressive nuance, as described by the dynamic attention theory put forth by Large and Jones^43^. Complicating the picture further, these structures of speech are thought to coordinate with internal (neurobiological) rhythms in the listener to generate temporal predictions, creating a “complex rhythmic flow pattern”^43^ that helps tether the attention of speaker and listener. This complex attentional tethering is indexed by interpersonal pupillary synchrony^28,30,53^.

Exactly which external (speech) and internal (prediction, attention) features contribute to pupillary synchrony between speakers and listeners is not well understood. Pupil dilations have been used to adjudicate to which *piece* of music someone is attending given two alternatives, but not which auditory features, cognitive processes, or likely combinations of features and processes, are causing those dilations^2^. Pupil dilations reflect aggregate responses from multiple brain regions and attention networks^54^ underpinning multiple cognitive processes across multiple timescales^2^. It is beyond the scope of the present paper to identify which auditory features, cognitive processes (or, likely, complex aggregates of both) are reflected in the pupillary time series recorded here. Our question is simply whether interpersonal pupillary synchrony, a naturally varying corollary of shared attention, is predicted by entrainment synchrony.

Many studies have investigated how people synchronize to external events in the environment^17,55^, and some have also studied pupillary synchrony between individuals^28–30^, but none have yet drawn a connection between the two. The finding here—that people who entrained more strongly to rhythmic tones were also more strongly synchronized with a storyteller—suggests that both forms of synchrony may be rooted in a common faculty. We note that this finding is not mutually exclusive with differences between these forms. For example, cerebellar lesion patients have difficulty representing the timing of salient events leading to deficits in synchronized tapping yet unimpaired performance on more continuous movement tasks^56^.

Research on interpersonal synchrony has tended to link synchrony with positively-valenced and societally beneficial outcomes such as social bonding, shared beliefs, and the cultural transmission of information^57–62^. However, it is important to note that being strongly coupled to an external stimulus may not always be optimal^63,64^. For example, synchrony can lead to poor affective regulation in dyads^65^ as well as deindividuation and a “mob mentality” in crowds (e.g., soccer crowds chanting in unison ^66^;). Conversely, decoupling from external stimuli may promote individuation and self-reflection^67^. Valenced effects of synchrony also depend on *what* is being synchronized. Couples whose cortical levels synchronized due to shared physiological arousal also shared in the associated negative health effects of high cholesterol^68^. Both coupling and decoupling have costs and benefits depending on context, and flexibly moving between both states may be optimal for many phenomena (e.g., creativity^69^; social engagement^30^).

Given both the benefits and costs of synchronization, it is possible that individual variation in the tendency to synchronize has collective benefits. Many social species solve complex problems by harnessing individual variability for collective ends. Social insects, for example, rely on inter-individual variation in stress thresholds to produce an effective, collective response to environmental threats^70,71^. Social interaction, a key feature of human intelligence, affords the distribution of knowledge and computation in ways that minimize individual effort and redundancy while maximizing access to diverse expertise^72,73^. Note that we find inter-individual variation in an individual’s *tendency* to synchronize—not whether an individual synchronizes at all. Previous work has identified that even when a listener synchronizes very little with a speaker overall, they still synchronize occasionally, and these moments are not random but predicted by emotional salience^28^. That is, given a strong enough attention-driving stimulus, even low synchronizers (i.e., those who have a high-threshold for synchronizing) will “tune in.” This variability in synchrony thresholds may be particularly useful in the collective. Ready (low threshold) synchronizers may increase social bonding in a group, while reluctant (high threshold) synchronizers may help limit contagion. As is generally true across psychological science, more research is needed to assess how individual variation influences multi-individual phenomena.

Much of the literature on attention relies on experiments that use static paradigms such as visual arrays. This research is unable to fully account for how we attend to the kind of dynamic, naturally varying phenomena that populate our world, including those produced by other people^55^. Because of these methodological constraints, how our attention dynamically couples with events and other minds in ways that have downstream consequences for perceptual, cognitive, and social phenomena (e.g., information processing, socioemotional regulation) is not well understood.

The current studies add to a small but growing scientific literature that uses temporally sensitive methods to study attentional dynamics across contexts. Specifically, we build on previous work showing that pupils synchronize with attended isochronous rhythms^18^ as well as more complex, naturally varying temporal dynamics^28^. We find evidence for a robust individual difference in a global tendency to synchronize. People who tend to synchronize to rigidly periodic beats also synchronize to the naturally varying dynamics associated with another person’s attentional state. This individual difference is consistent with a common underlying faculty underpinning these diverse forms of synchrony.

## Supplementary Information

Supplementary Information.

## Data Availability

All pupillometry and behavioral responses associated with this manuscript have been made available at https://github.com/sophiewohltjen/individual-attention.

## Code availability

All custom scripts associated with this manuscript have been made available at https://github.com/sophiewohltjen/individual-attention.
