# Supplementary material for: Synchrony to a beat predicts synchrony with other minds
Source: Sci Rep. 2023 Mar 3;13:3591. doi: 10.1038/s41598-023-29776-6 (PMC9984464; doi:10.1038/s41598-023-29776-6)
Supplement: Supplementary file 1 — Supplementary Information. [file 41598_2023_29776_MOESM1_ESM.pdf]

Supplementary information for
Synchrony to a beat predicts synchrony with other minds

Sophie Wohltjen, Brigitta Toth, Adam Boncz, and Thalia Wheatley

Determining individual decibel thresholds for target values in the auditory oddball paradigm

We determined how much to lower the decibel level for target tones on an individual basis, using a transformed up-down staircase procedure from the PSYCHOACOUSTICS toolbox in Matlab (Soranzo & Grassi, 2014). For this, participants listened to sets of three tones and determined which was the loudest. The loudest tone started at 10 decibels higher than the other two tones in the sequence. If the participant correctly identified the loudest tone twice, the algorithm then began working down a *staircase* of decreasing decibel differences, going down one *step* each time the participant correctly identified two tones at that decibel level, and increasing one *step* each time the participant incorrectly identified the loudest tone at that decibel level. Each block continued until the participant hit 12 *reversals*, or changes from going down the staircase to going up the staircase. There were three total blocks. Decibel levels decreased by a factor of two (e.g. 10 decibels to 5 decibels) for the first four reversals, then decreased by a factor of 1.4 (e.g. 10 decibels to 7.14 decibels) for the final eight reversals. The final threshold was then calculated by averaging the decibel levels at the last eight reversals, then averaging these over the all three participation blocks. We then applied this threshold to our target tones.

Reliable differences in individual pupil responses are present in all four testing conditions

To determine whether participants reliably differed in their tendency to entrain their continuous pupillary fluctuations to the rhythmic interval of the tones in the oddball task, we established that participants' pupils reliably responded to our standard and target conditions, but we also investigated the reliability of participants' pupil responses to novel and omission conditions. To do this, we tested the similarity of their averaged pupil responses in each of these two trial-types of the oddball experiment across testing sessions. Pearson correlations compared participants' pupil response curves in each session to their own responses from different sessions. We then compared the resulting R values from those correlations to the R values obtained by correlating pupil responses between participants. Because our data was not normally distributed (within-

participant correlations were skewed toward higher R values) and because we had more between-participants observations than within-participants observations, we used the non-parametric Mann-Whitney U test to compare pupil response similarity within and between participants.

Participants' pupil response curves to novel tones ($U=42812$, $p < 0.001$, cohen's $d = 0.82$) and omission tones ($U=49963$, $p < 0.001$, cohen's $d = 0.69$) were more similar to themselves across sessions than compared to other participants, further supporting the idea that an individual's pupillary fluctuations reflect a stable individual difference throughout the oddball task.

Determining which features of the individual pupillary response to relate to task performance and task entrainment

We chose two features of individuals' pupil response curves to novel and omission trials—the maximum amplitude of their pupil response and the time their pupil took to reach this maximum amplitude (tmax)—to relate to potential differences in entrainment synchrony and task performance. These two features were selected based on Hoeks & Levelt's (1993) paper modeling attention-related pupil responses as impulse response functions triggered by attentional pulses. According to Hoeks and Levelt (1993), amplitude and tmax are two of three parameters that characterize the shape of pupil responses. The third free-varying parameter in their model accounted for the additive nature of attention pulses. This third feature did not pertain to the current study due to the spacing of trials and is not included here.

Individual pupillary responses to target tones predict oddball detection and entrainment synchrony

Because we compared pupillary responses to omission and novel tones to task entrainment and task performance, we wondered whether these individual pupil responses to target and standard tones were also predictive of task performance and task entrainment synchrony. To test whether pupillary responses predicted task performance (as measured by *d* prime) and task entrainment synchrony, we ran two linear regressions predicting 1) participants' *d* prime scores and 2) participants' entrainment synchrony scores from two features of their pupil response curve, per

condition—the maximum amplitude of their pupil response and the time their pupil took to reach this maximum amplitude (tmax).

We found significant relationships between individual pupillary responses during target conditions and 1) oddball task performance ($f(3,68) = 5.21$, $p < 0.005$, adjusted $R^2 = 0.15$) and 2) entrainment synchrony ($f(3,68) = 7.32$, $p < 0.001$, adjusted $R^2 = 0.21$). Target pupil response amplitude positively predicted task performance (target detection sensitivity, measured by *d* prime; $\beta = 0.39$, FDR corrected $p < 0.005$) and entrainment synchrony ($\beta = 0.49$, FDR corrected $p < 0.001$), such that the greater the dilation, the better the participant performed on the oddball task and the more they were entrained to the task structure. target pupil response tmax inversely predicted task performance ($\beta = -0.29$, FDR corrected $p = 0.04$), such that the faster participants' pupils dilated, the better they performed. There was no relationship between target tmax and entrainment synchrony. The relationships between target amplitude, target tmax, and task performance are illustrated in supplementary figures 1a and 1b.

We did not find that pupil responses to standard trials predicted task performance ($f(3,68) = 1.42$, FDR corrected $p = 0.3$, adjusted $R^2 = 0.02$) or entrainment synchrony ($f(3,68) = 0.37$, FDR corrected $p = 0.77$, adjusted $R^2 = -0.02$). This was initially surprising, since we do find that entrained pupillary fluctuations at the interval of the standard tone does predict task performance. However, we noticed that participants' pupil responses to standard trials were on average much smaller than their responses during all of the other conditions (see supplementary figure 2). Because standard tones occurred predictably and frequently throughout the task, and because participants were not directly instructed to attend to standard tones, this result could simply be due to the muted response to these tones across participants.

Individual pupillary responses to target tones predict pupillary synchrony during storytelling

Because we found relationships between pupil responses to target trials and entrainment synchrony, we wondered whether responses to these trials were also predictive of individuals' pupillary synchrony with a storyteller. A linear mixed effects model examining the relationship between speaker-listener pupillary synchrony and individual pupillary responses during target

conditions was also significant. target pupil response amplitude positively predicted speaker-listener pupillary synchrony ($t(59.6) = 3.4$, $\beta = 0.35$, $p < 0.005$), such that the greater the target dilation during the oddball task, the more synchrony with a speaker telling a story. target pupil response tmax inversely predicted speaker-listener pupillary synchrony ($t(60.1) = -3.2$, $\beta = -0.31$, $p < 0.005$), such that the faster participants' pupils dilated during the oddball task, the more synchrony with a speaker telling a story. These relationships are illustrated in supplementary figures 1c and 1d.

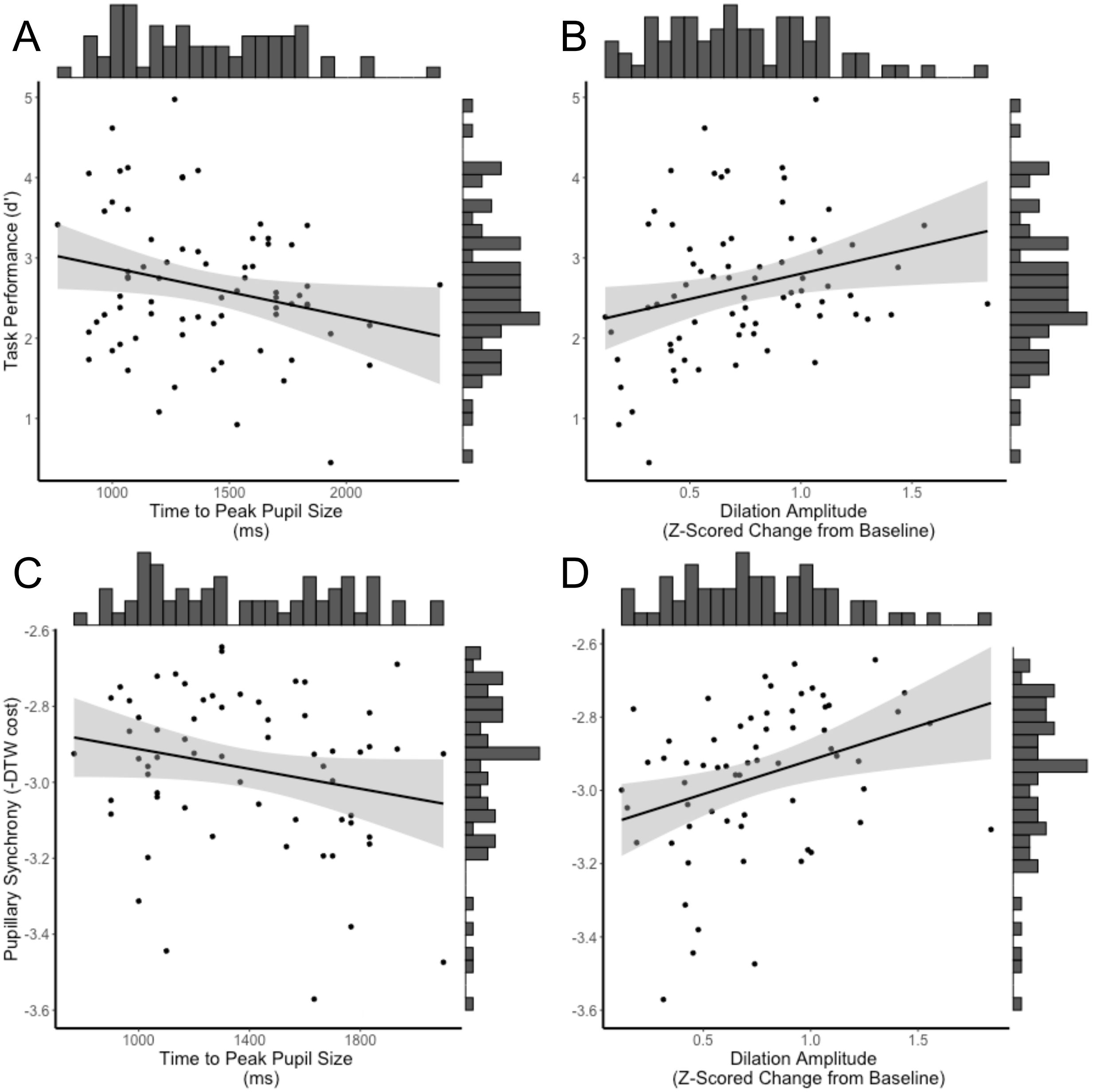

Supplementary Figure 1. Data and estimated slopes for the relationship between 1) task performance and **A)** time to peak pupil size (tmax) and **B)** amplitude, and 2) pupillary synchrony between speakers and listeners with **C)** tmax of listeners' attention related pupil responses during the oddball task and **D)** amplitude of listeners' attention related pupil responses during the oddball task. Tmax was negatively related to both task performance and pupillary synchrony such that the quicker participants reached peak pupil dilation, the better they performed on the oddball task and the more synchronous they were with a speaker telling stories. Amplitude was positively related to task performance and pupillary synchrony such that the larger participants' attention-related pupil responses were, the better they performed on the oddball task and the more synchronous they were with a speaker telling stories.

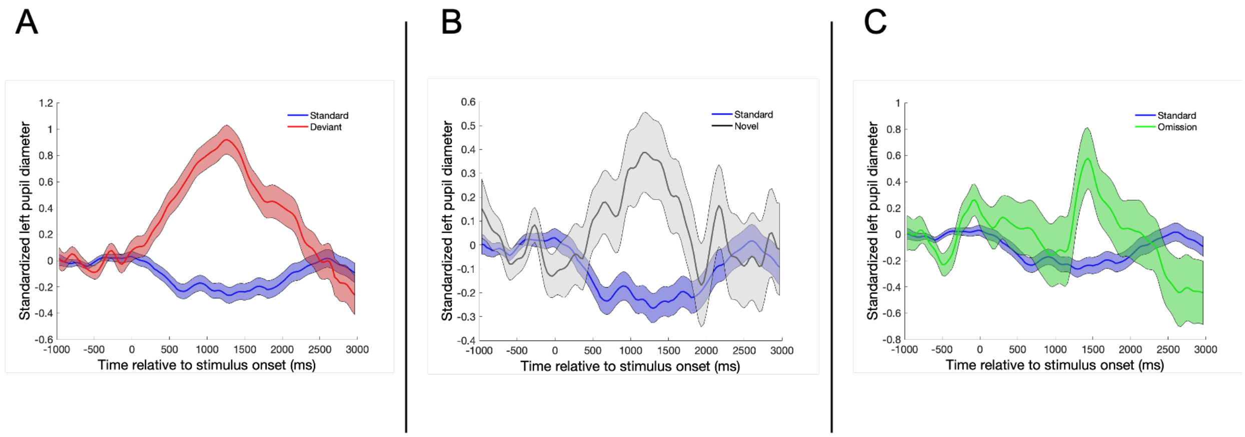

Supplementary Figure 2. Illustration of attention-related pupil responses from a single participant for **A)** target trials, **B)** novel trials, and **C)** omission trials. Pupil responses to standard trials are plotted for comparison in all three figures.

Testing the relationship between individual differences in pupillary correlates of attention and reaction time in the auditory oddball paradigm

We found that individuals' attention-related pupil responses and their ability to entrain to the oddball task structure both predicted their target detection performance during the oddball task. We wondered whether participants' reaction times when identifying target tones would show a

similar relationship. We ran linear regressions predicting participants' reaction time from their trial-level pupil responses and from their task entrainment synchrony. This model was significant for pupil responses to the target condition ($f(3,67) = 7.88$, $p < 0.001$, adjusted $R^2 = 0.23$). This model fit was driven by tmax, which positively predicted reaction time ($\beta = 0.45$, FDR corrected $p < 0.001$), such that the quicker participants reacted to the target, the quicker their pupils dilated. Amplitude did not significantly relate to reaction time ($\beta = -0.008$, FDR corrected $p = 0.94$). While omission trials did significantly predict reaction time ($f(3,67) = 2.85$, $p = 0.04$, adjusted $R^2 = 0.07$), no main effects survived FDR correction. There were also no significant effects associated with novel trials ($f(3,67) = 1.17$, FDR corrected $p = 0.43$, adjusted $R^2 = 0.007$), standard trials ($f(3,67) = 0.15$, FDR corrected $p = 0.94$, adjusted $R^2 = -0.04$), or entrainment power ($f(1,69) = 2.95$, FDR corrected $p = 0.24$, adjusted $R^2 = 0.03$) predicting reaction time to target events. These results could be due to participants with quick reaction times but low task performance, who may be quick to respond *incorrectly*.

References

Hoeks, B., & Levelt, W. J. M. (1993). Pupillary dilation as a measure of attention: a quantitative system analysis. *Behavior Research Methods, Instruments, & Computers: A Journal of the Psychonomic Society, Inc*, *25*(1), 16–26.

Soranzo, A., & Grassi, M. (2014). PSYCHOACOUSTICS: a comprehensive MATLAB toolbox for auditory testing. *Frontiers in Psychology*, *5*, 712.
